# Stigmatizing attitudes about lung cancer among individuals who smoke cigarettes

**DOI:** 10.18332/tid/146907

**Published:** 2022-04-14

**Authors:** Destiny Diaz, Amanda J. Quisenberry, Brian V. Fix, Christine E. Sheffer, Richard J. O'Connor

**Affiliations:** 1 Department of Health Behavior, Roswell Park Comprehensive Cancer Center, Buffalo, United States

**Keywords:** lung cancer, smoking cessation, tobacco, smoking, stigma

## Abstract

**INTRODUCTION:**

Many individuals with lung cancer report experiencing stigma associated with their diagnosis. The objective of this study was to explore how different factors, including smoking status, lung cancer concern, and thoughts on smoking behaviors, were associated with increased stigmatizing attitudes towards people with lung cancer.

**METHODS:**

In 2015, a Web-based survey was completed by people who currently smoked. Participants (n=1419) aged 18–65 years were randomly assigned to one of three scenarios in which the character who was diagnosed with lung cancer currently, formerly, or never smoked cigarettes. Two aspects of stigma were assessed: blaming the victim and negative attributions about people with lung cancer.

**RESULTS:**

For blaming the victim and negative attributions, lung cancer stigma differed by scenario (described smoking status, p<0.0001), when adjusting for race, sex, education level, age, income, nicotine dependence, quit intentions, and quit attempts. Higher levels of lung cancer concern were associated with greater blaming the victim (p=0.001), when adjusting for scenario and other significant correlates.

**CONCLUSIONS:**

The findings suggest that stigmatizing attitudes from people who smoke towards people with lung cancer may be reflective of how they feel about their own smoking habits. We suggest that specific messaging guidelines that avoid an over emphasis on an individual’s smoking status, cessation interventions that address stigma, and screening messages tailored to smoking status, may help to lessen the burden of lung cancer stigma.

## INTRODUCTION

Cigarette smoking is the primary risk factor for lung cancer^1^. It has become denormalized in many countries, with approaches including smoke-free air laws, media campaigns, pictorial health warnings on tobacco products, and anti-smoking policies such as prohibiting the hiring of people who smoke or requiring higher health insurance premiums for people who smoke^2^. In this denormalization process, smoking and its associated effects have begun to become stigmatized^3-5^. Denormalization efforts (aimed at discouraging tobacco use in order to decrease the associated negative health effects) has contributed to the decrease of smoking but may have also yielded other consequences, such as the psychological burden of living with a stigmatized, tobacco-related disease such as lung cancer.

Goffman^6^ described stigma as ‘the situation of the individual who is disqualified from full social acceptance’. Lung cancer stigma includes the explicit and/or implicit belief that the condition was preventable and thus blame falls on the patient because they smoked cigarettes^7^. Stigma attached to an illness such as lung cancer can lead to discrimination^8^, depression, reduced quality of life^9^, and delays in treatment-seeking^10^. Because of lung cancer’s strong link to smoking, stigma represents a psychosocial barrier for people with lung cancer, regardless of their smoking status^11^. Literature confirms the existence of lung cancer stigma^12^, its effects on clinical care^11,13,14^, its physiological effects^15^, ways to measure it^16,17^, and interventions to prevent it^18^. Research has also demonstrated that non-smoking respondents tend to stigmatize people with lung cancer, especially those who smoke^19^. Understanding lung cancer stigma, especially among individuals experiencing these attitudes, might help to inform strategies to mitigate the impact of lung cancer stigma on individuals who smoke.

### Theoretical framework

This study follows the work by Bresnahan et al.^19^ which indicated that respondents who did not smoke tended to stigmatize people with lung cancer, especially people who smoked who developed lung cancer, and that respondents who smoked showed greater sympathy for people with lung cancer. Their results also demonstrated that people with lung cancer who also smoked received more blame, compared to people who did not smoke. Their theoretical framework addressed concepts such as victim blaming, negative attributions (selfishness, impulsivity, low will power), controllability of behavior, and smoking cessation efficacy (based on the Self-Administered Nicotine Dependence Scale^20^). Bresnahan et al.^19^ hypothesized that controllability of smoking and smoking cessation efficacy would be related to blame and negative attributions about people with lung cancer. They noted that ‘when controllability over the negative effects of a disease is perceived as high, more blame and negative attributions are likely to be assigned’. Furthermore, if a person believes that cessation treatments (such as prescription medicines) are always effective for everyone, they may believe it is easy for a person who smokes to quit. Therefore, these individuals may stigmatize people who smoke who also get lung cancer because they did not quit early enough to prevent lung cancer from developing.

How people who currently smoke stigmatize people with lung cancer is of interest in this study. They may assign blame to lung cancer survivors differently from people who do not smoke. Individuals who smoke use products with nicotine and most become addicted^21^. Thus, they may better understand how difficult it is to quit relative to those who do not smoke and may stigmatize those who formerly smoke and develop lung cancer less than those who currently smoke and who have lung cancer. Tobacco users experience markers of self-stigma such as self-loathing and shame related to their tobacco use^22^. Individuals who currently smoke have been shown to be concerned more and perceive their lung cancer risk as higher compared to those who formerly smoked^23^, possibly due in part to knowledge that cessation lowers the risk of many smoking-related diseases^24^. With higher lung cancer risk perception, these people who currently smoke know the consequences of their own smoking habits and thus their stigmatization of people with lung cancer may reflect their internal feelings of risk.

The difficulty many people who smoke cigarettes face in maintaining abstinence from smoking can be underappreciated. Many environmental and physiological circumstances contribute to smoking cessation, but popular beliefs about free choice may lead to stigmatization of people who smoke (more than people who do not smoke). Perceived stigma has been found to be higher among people with lung cancer who currently smoke compared to people who formerly or never smoked^25^. Nicotine is highly addictive^21^ and attenuates the ability to effectively make decisions regarding behavior change. Individuals who believe that smoking cessation will greatly reduce lung cancer risk and/or quit smoking themselves, may stigmatize people who smoke for not quitting. The stigma-ridden zeitgeist may change towards people with lung cancer if it is known that a person with lung cancer had quit successfully.

This study provides a different perspective from existing literature on lung cancer stigma. We examine whether or not the described smoking status of a person with lung cancer has an impact on stigmatizing attitudes toward these individuals. We specifically examine stigmatization by individuals who smoke cigarettes and explore whether or not this stigma reflects their internal feelings about lung cancer.

**Figure 1:**
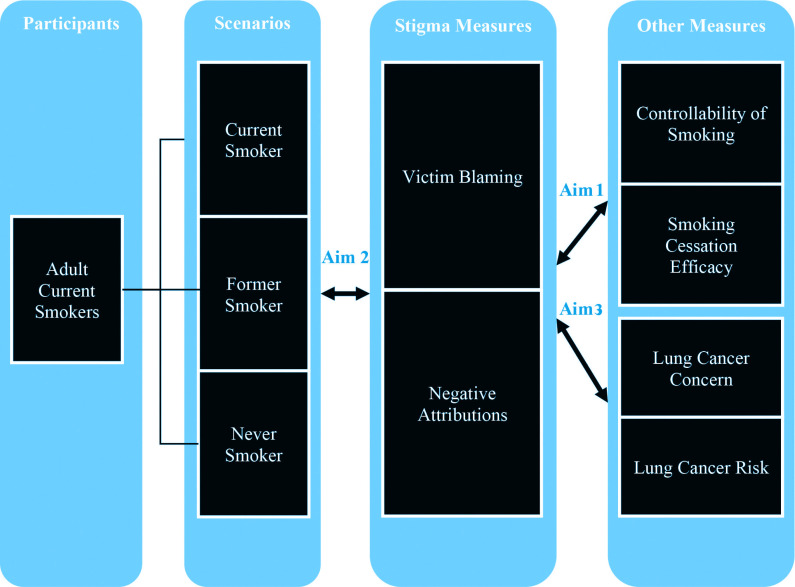
Theoretical framework of analysis and research aims

The respondents were individuals who currently smoked and they were given scenarios about a person with lung cancer who was either a current, former, or never smoker. Of interest was how much they would stigmatize people with lung cancer. Stigma comprised two different concepts: victim blaming, and negative attributions about people with lung cancer. Other measures included smoking cessation efficacy, controllability of smoking, lung cancer concern, and perceived lung cancer risk (Figure 1).

The implications of this study include informing strategies that can minimize the burden of lung cancer stigma. Our aims are:

1. Determine the relationship between the stigma measures and perceived efficacy of smoking cessation/controllability of smoking.
2. Determine the relationship between the stigma measures and scenario.
3. Determine the relationship between the stigma measures and lung cancer concern/perceived risk.

## METHODS

### Sample

Participants were 1419 aged 18–65 years who currently smoked cigarettes, daily or non-daily, and smoked more than 100 cigarettes in their lifetime. Participants were a subset of respondents in a larger survey conducted in 2015 among 3001 US residents aged 14–65 years, recruited from existing Web panels (Global Market Insite; GMI). All participants appropriately consented and were compensated 60 GMI marketpoints (US$3) for participation.

### Measures

*Demographics*

The demographics of interest were race/ethnicity, sex, age, education level, and income.

*Nicotine dependence*

Nicotine dependence was measured using The Heaviness of Smoking Index (HSI). The HSI uses two questions from the Fagerström Tolerance Questionnaire and the Fagerström Test for Nicotine Dependence: time to first smoke in the morning and number of cigarettes per day^26^.

*Lung cancer concern*

The question ‘How often do you worry about getting lung cancer? Would you say’ was asked of participants. Response options provided were ‘rarely or never; sometimes; often; or all of the time’. This variable was used to explore Aim 3 (determining the relationship between the stigma measures and lung cancer worry (concern) /perceived risk).

*Perceived lung cancer risk*

This question asked participants to rate their answer to the following question: ‘Compared to other smokers your age, what do you think your chances are of getting lung cancer?’. The answers were scaled from 1–5, where 1 represented ‘much less risk’ and 5 represented ‘much more risk’. In order to limit the number of groups, this variable was recoded into a 1–3 scale: 1 representing ‘less risk’ (responses 1–2), 2 representing ‘same risk’ (response 3), or 3 representing ‘higher risk’ (responses 4–5). This variable was used to explore Aim 3 (determining the relationship between the stigma measures and lung cancer concern/perceived risk).

*Quit intentions*

To determine if a participant was planning on quitting, the responses to the following question were analyzed: ‘Are you seriously thinking of quitting smoking?’. The possible responses were ‘Yes, within the next 30 days’, ‘Yes, within the next 6 months’ and ‘No, not thinking of quitting’.

*Past year quit attempts*

In order to assess how many times a respondent has tried to quit smoking within the last year, the responses to the following question were used: ‘In the last year, how many times have you quit smoking for at least 24 hours?’. The responses were then recoded into the following 3 categories: 0, 1–2, and ≥3 times.

*Perceived efficacy of smoking cessation*

Smoking cessation efficacy comprised 9 questions (α=0.90) rated on a 5-point Likert-type scale where 1 was ‘strongly disagree’ and 5 was ‘strongly agree’ (questions described elsewhere^19^).

Sample items included: ‘Chewing nicotine gum is an effective way to quit smoking’. In order to compare this measure across different groups, we summed the scores of the questions in this construct for each participant. An average variable was created by dividing this summed score by the number of questions in this construct. A higher mean score indicated a higher amount of this measure.

*Controllability of smoking*

Controllability of smoking comprised 6 questions (α=0.78) rated on a 5-point Likert-type scale where 1 was ‘strongly disagree’ and 5 was ‘strongly agree’ (questions described elsewhere^19^).

Sample items included: ‘Smoking is a behavior which can be controlled’. This measure was calculated in a manner similar to the way smoking cessation efficacy was.

*Victim blaming and negative attributions about people with lung cancer*

The stigma measures used in this analysis were victim blaming and negative attributions about people with lung cancer. These comprised multiple questions rated on a 5-point Likert-type scale where 1 was ‘strongly disagree’ and 5 was ‘strongly agree’ (questions described elsewhere^19^). Blaming the victim comprised 5 questions (α=0.92) and sample items included: ‘It is clear that this man could have prevented his cancer’. Negative attributions about people with lung cancer comprised 5 questions (α=0.95) and sample items included: ‘If smokers used nicotine gum, they should be able to quit smoking’. These measures were calculated in a manner similar to the way smoking cessation efficacy was.

### Experimental methods

Participants were randomly assigned to one of three scenarios^19^ in which the character with a cancerous tumor in the lung currently smokes (has been a heavy smoker since high school), formerly smoked (had been a heavy smoker, but quit about 10 years ago), or never smoked. After reading the scenario, participants were asked to complete a series of questions regarding smoking cessation efficacy, controllability of smoking, and the 2 stigma constructs: blaming the victim, and negative attributions about people with lung cancer.

### Statistical analyses

Data were analyzed using SPSS Statistics 25 (IBM, Armonk NY). Significance was evaluated at the α=0.05 level. For descriptive statistics of participant characteristics, chi-squared tests for associations were used to provide the distribution of each participant measure and demographic by scenario. Standardized and adjusted residuals were calculated in order to provide insight on where any significant differences found may be located. One-way ANOVA tests were used to test unadjusted mean differences for each stigma construct by scenario. For scenarios, Bonferroni *post hoc* tests were also used to determine where these differences were. One-way ANOVA tests were used to evaluate if there were differences in the stigma measures by varying levels of demographics and these significant correlates were included in the models testing the relationships between the stigma measures and scenario, lung cancer concern, and lung cancer perceived risk.

Our dependent variables were the stigma measures (victim blaming and negative attributes) because we examined how lung cancer stigma was affected by different factors. These factors include participants’ thoughts on cessation efficacy, controllability of smoking, and lung cancer concern/perceived risk; thus, these were our independent variables. Furthermore, we were also interested in how the smoking status of the person with lung cancer impacted stigma, thus another independent variable was scenario. More specifically:

- To investigate Aim 1 (determining the relationship between the stigma measures and perceived efficacy of smoking cessation/controllability of smoking), non-parametric correlations, specifically Spearman’s rho (ρ), were used to determine if victim blaming and negative attributions about people with lung cancer were related to smoking cessation efficacy and controllability of smoking.
- To investigate Aim 2 (determining the relationship between the stigma measures and scenario), univariate general linear models were used to evaluate differences in the stigma measure means (dependent variables) by scenario (independent variable), adjusting for the identified significant correlates.
- To investigate Aim 3 (determining the relationship between the stigma measures and lung cancer concern/perceived risk), univariate general linear models were used to evaluate differences in the stigma measure means (dependent variables) by levels of lung cancer concern/perceived risk (independent variables), adjusting for the identified significant correlates.

## RESULTS

### Participant characteristics

Participants included 1419 individuals who smoked daily (86.0%) or non-daily (14.0%). Majority of the sample consisted of males (56.3%), individuals aged 26–35 years (40.9%), those who had some college/ technical school (34.9%), and those who were White (74.7%). Table 1 describes the demographic/ behavioral correlate breakdown of participant characteristics by scenario with the paramount standardized residuals noted. There were significant differences found within education level (p=0.005), income (p=0.029), and lung cancer concern (p=0.010).

**Table 1:** Participant demographics by scenario, US residents recruited from existing Web panels, 2015 (N=1419)

*Characteristics*	*Characteristics Total n (%)*	*Current smoker scenario n (%)*	*Former smoker scenario n (%)*	*Never smoker scenario n (%)*	p* χ^2^
**Overall**	1419 (100)	463 (32.6)	469 (33.1)	487 (34.3)	
**Race**					0.285 11.997
White	1060 (74.7)	354 (76.5)	349 (74.4)	357 (73.3)	
Black	68 (4.8)	25 (5.4)	21 (4.5)	22 (4.5)	
Asian/ Pacific Islander	33 (2.3)	11 (2.4)	12 (2.6)	10 (2.1)	
American Indian/Alaskan Native	10 (0.7)	1 (0.2)	7 (1.5)^⁋^	2 (0.4)	
Hispanic	238 (16.8)	71 (15.3)	75 (16.0)	92 (18.9)	
Other	10 (0.7)	1 (0.2)	5 (1.1)	4 (0.8)	
**Sex**					0.641 0.889
Male	802 (56.5)	264 (57.0)	257 (54.8)	281 (57.7)	
Female	617 (43.5)	199 (43.0)	212 (45.2)	206 (42.3)	
**Education level**					**0.005** 21.752
Did not complete HS	32 (2.3)	8 (1.7)	10 (2.1)	14 (2.9)	
HS Grad/GED	227 (16.0)	82 (17.7)	81 (17.3)	64 (13.1)^‡^	
Some college/Technical/Associates	495 (34.9)	152 (32.8)	188 (40.1)^⁋^	155 (31.8)	
Bachelor’s degree	478 (33.7)	159 (34.3)	146 (31.1)	173 (35.5)	
Master’s/Doctorate	187 (13.2)	62 (13.4)	44 (9.4)^‡^	81 (16.6)^⁋^	
**Age** (years)					0.585 6.561
18–25	55 (3.9)	14 (3.0)	21 (4.5)	20 (4.1)	
26–35	581 (40.9)	187 (40.4)	182 (38.8)	212 (43.5)	
36–45	337 (23.7)	118 (25.5)	106 (22.6)	113 (23.2)	
46–55	251 (17.7)	78 (16.8)	95 (20.3)	78 (16.0)	
≥56	195 (13.7)	66 (14.3)	65 (13.9)	64 (13.1)	
**Smoking status**					0.152 3.770
Daily	1220 (86.0)	409 (88.3)	402 (85.7)	409 (84.0)	
Non-daily	199 (14.0)	54 (11.7)	67 (14.3)	78 (16.0)	
**Income** (US$)					**0.029** 20.056
≤25000	186 (13.1)	53 (11.4)	77 (16.4)^⁋^	56 (11.5)	
25001–50000	262 (18.5)	91 (19.7)	80 (17.1)	91 (18.7)	
50001–75000	278 (19.6)	91 (19.7)	90 (19.2)	97 (19.9)	
75001–100000	309 (21.8)	100 (21.6)	108 (23.0)	101 (20.7)	
≥100001	373 (26.3)	128 (27.6)	106 (22.6)^‡^	139 (28.5)	
Prefer not to answer	11 (0.8)	0 (0.0)^‡^	8 (1.7)^⁋^	3 (0.6)	
**Lung cancer concern**					**0.010** 16.818
Rarely or never	299 (21.1)	102 (22.0)	114 (24.3)^⁋^	83 (17.0)^‡^	
Sometimes	677 (47.7)	221 (47.7)	209 (44.6)	247 (50.7)	
Often	324 (22.8)	115 (24.8)	100 (21.3)	109 (22.4)	
All of the time	119 (8.4)	25 (5.4)^‡^	46 (9.8)	48 (9.9)	
**Comparative lung cancer risk**					0.228 5.642
Less	311 (21.9)	99 (21.4)	99 (21.1)	113 (23.2)	
Same	684 (48.2)	235 (50.8)	235 (50.1)	214 (43.9)^‡^	
More	424 (29.9)	129 (27.9)	135 (28.8)	160 (32.9)	
**Heaviness of smoking**					0.182 3.405
Low dependence	1108 (78.1)	351 (75.8)	364 (77.6)	393 (80.7)	
High dependence	311 (21.9)	112 (24.2)	105 (22.4)	94 (19.3)	
**Quit intentions**					0.179 6.285
Within the next 30 days	379 (26.7)	132 (28.5)	106 (22.6)^‡^	141 (29.0)	
Within the next 6 months	561 (39.5)	178 (38.4)	193 (41.2)	190 (39.0)	
Not thinking about quitting	479 (33.8)	153 (33.0)	170 (36.2)	156 (32.0)	
**Past year quit attempts**					0.371 4.268
0	495 (34.9)	168 (36.3)	173 (36.9)	154 (31.6)	
1–2	386 (27.2)	118 (25.5)	129 (27.5)	139 (28.5)	
≥3	538 (37.9)	177 (38.2)	167 (35.6)	194 (39.8)	

* Bold indicates statistical significance.

‡ Adjusted standardized residuals < -2.0.

⁋ Adjusted standardized residuals > 2.0.

### Descriptive statistics

For smoking cessation efficacy and controllability of smoking, the unadjusted overall means and standard deviations were 3.38±0.81 and 3.86±0.67, respectively. For the stigma measures, victim blaming and controllability of smoking, the unadjusted overall means were 3.43±1.07 and 2.99±1.17, respectively. Breaking up victim blaming scores by scenario, the mean score was highest for the scenario in which the person smoked, followed by the scenarios where the person formerly smoked and never smoked: 3.74±0.86, 3.64±0.89 and 2.93±1.23. There was a statistically significant difference between scenarios within victim blaming as determined by one-way ANOVA [F(2, 1416)=91.37, p<0.0001] and 11.4% of the variance in victim blaming was accounted for by scenario. A Bonferroni *post hoc* test revealed that stigma scores were significantly higher (p<0.0001) for the scenario where the person formerly/currently smoked compared to the scenario where the person never smoked. For negative attributions, the mean score was highest for the scenario where the person formerly smoked, followed by the scenarios where the person currently smoked and never smoked: 3.08±1.12, 3.06±1.12 and 2.84±1.26. Without adjusting, there was a statistically significant difference between scenarios within negative attributions [F (2, 1416)=6.33, p=0.002] and 0.9% of the variance was accounted for by scenario. Stigma scores were significantly higher for the scenario where the person with lung cancer currently/formerly smoked compared to the scenario where the person never smoked (p=0.009 and p=0.005, respectively).

### Identifying differences in lung cancer stigma by demographic/behavioral correlates

For victim blaming, significant differences in means were found between the different categories of race (p<0.0001), sex (p<0.0001), education level (p<0.0001), age (p<0.0001), income (p<0.0001), nicotine dependence (p<0.0001), quit intentions (p<0.0001), and quit attempts (p<0.0001). Those who: identified as Asian/Pacific Islander (3.76±0.69), were male (3.58±1.01), had a graduate degree (3.64±1.01), were aged 18–34 years (3.71±0.96), had an income ≥$100001 (3.78±0.97), had low nicotine dependence (3.48±1.04), planned to quit within the next 30 days (3.83±1.00), and had attempted to quit ≥3 times within the past year (3.72±0.98), had the highest blaming the victim means. These variables were used as covariates in the analysis involving the victim blaming construct.

For negative attributions about people with lung cancer, significant differences in means were found between the different categories of race (p<0.0001), sex (p<0.0001), education level (p<0.0001), age (p<0.0001), income (p<0.0001), nicotine dependence (p<0.0001), quit intentions (p<0.0001), and quit attempts (p<0.0001). Those who: identified as Hispanic (3.57±1.06), were male (3.21±1.14), had a graduate degree (3.641±0.21), were aged 18–34 years (3.40±1.15), had an income ≥$100001 (3.53±1.13), had low nicotine dependence (3.12±1.15), planned to quit within the next 30 days (3.68±1.08), and had attempted to quit ≥3 within the past year (3.47±1.15) had the highest negative attributions about people with lung cancer means. These variables were used as covariates in the analysis involving the negative attributions about people with lung cancer construct.

### Aim 1: Determining the relationship between the stigma measures and perceived efficacy of smoking cessation/controllability of smoking

Smoking cessation efficacy was positively and moderately associated with victim blaming (ρ=0.533, p<0.0001) and positively and strongly associated with negative attributions about people with lung cancer (ρ=0.683, p<0.0001). Controllability of smoking was positively and moderately associated with victim blaming (ρ=0.446, p<0.0001) and positively associated with negative attributions about people with lung cancer, with a weak rho value (ρ=0.335, p<0.0001). Since the stigma measures were associated with smoking cessation efficacy and controllability of smoking, they were included as covariates (along with the other significant correlates found) in the models used to explore Aims 2 and 3 (determining the relationships between the stigma measures, scenario, and lung cancer concern/perceived risk).

### Aim 2: Determining the relationship between the stigma measures and scenario

**Figure 2:**
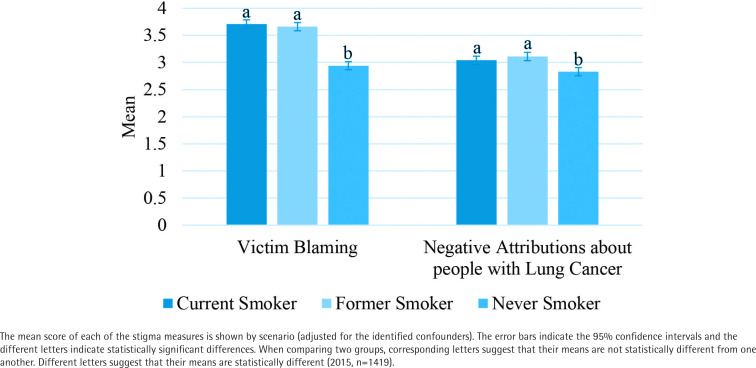
Stigma measures by scenario

For blaming the victim, there was a statistically significant difference between scenarios when adjusting for significant correlates found above [F(2, 1406)=125.367, p<0.0001]. The means were highest for the scenario where the person with lung cancer currently smoked followed by the scenarios where the person formerly/never smoked (mean±SE: 3.71±0.04; 3.66±0.04; 2.94±0.04, respectively). Bonferroni pairwise comparisons showed significant differences (p<0.0001) in means between the scenarios where the person never and formerly smoked and the scenarios where the person who never and currently smoked (Figure 2).

For negative attributions, there was a statistically significant difference between scenarios when adjusting for the significant correlates found above [F(2, 1406)=14.897, p<0.0001]. The means were highest for the scenario where the person formerly smoked followed by the scenarios where the person currently and never smoked (mean±SE: 3.11±0.04; 3.04±0.04; 2.83±0.04, respectively). Bonferroni pairwise comparisons show significant differences (p<0.0001) in means between the scenarios where the person with lung cancer never and formerly smoked and the scenarios where the person never and currently smoked (Figure 2). Since scenario was associated with the stigma measures, it was included as a covariate in the models used to explore Aim 3 (determining the relationship between the stigma measures and lung cancer concern/perceived risk).

### Aim 3: Determining the relationship between the stigma measures and lung cancer concern/ perceived risk

**Figure 3:**
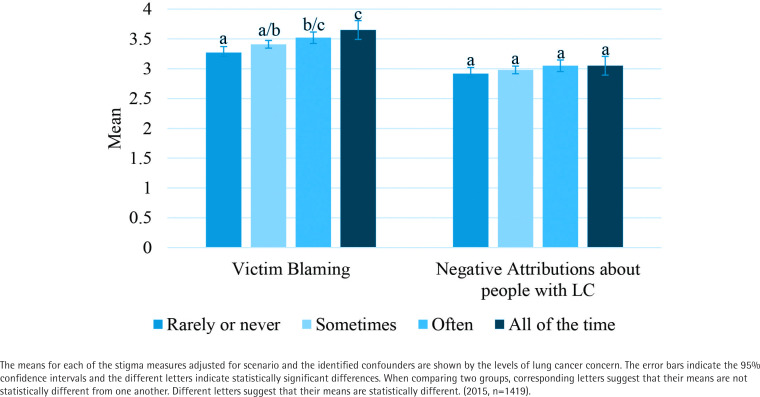
Stigma measures by lung cancer concern

For blaming the victim, there was a statistically significant difference between levels of lung cancer concern when adjusting for scenario and the other significant correlates found above [F(3, 1404)=5.914, p=0.001]. Higher lung cancer concern levels were associated with higher the blaming the victim means (mean±SE: 3.27±0.05; 3.41±0.03; 3.52±0.05; 3.65±0.08 for rarely or never, sometimes, often, and all of the time, respectively). Bonferroni pairwise comparisons show significant differences in means between rarely or never and often (p=0.005), rarely or never and all of the time (p=0.001), and sometimes and all of the time (p=0.041). For negative attributions, there was not a statistically significant difference between levels of lung cancer concern when adjusting for scenario and the significant correlates found above [F(3, 1404)=1.166, p=0.321] (Figure 3).

For blaming the victim, there was not a statistically significant difference between levels of perceived lung cancer risk when adjusting for scenario and the other significant correlates found above [F(2, 1405)=1.217, p=0.297]. For negative attributions, there was also not a statistically significant difference between levels of perceived lung cancer risk when adjusting for scenario and the other significant correlates found above [F(2, 1405)=0.339, p=0.713].

## DISCUSSION

This study examined how the smoking status of people described as having lung cancer impacted stigmatization by respondents who currently smoke. The results are similar to previous literature showing a significant main effect of described smoking status^19^. Perceived smoking cessation efficacy was positively associated with victim blaming and negative attributions about people with lung cancer (Aim 1), suggesting that the more a person believes that effective steps can be taken to quit, the more they will blame and assign negative attributions to people with lung cancer. Also, controllability of smoking was positively associated with victim blaming and negative attributions about people with lung cancer (Aim 1), suggesting that the more a person believes an individual can choose whether or not to smoke, the more they will blame and assign negative attributions to people with lung cancer. These results are consistent with what was found in the Bresnahan et al.^19^ study.

Different aspects of lung cancer stigma appear to be sensitive to the smoking status presented in the scenario (Aim 2). With regard to blaming the victim, levels were highest among those who received the scenario where the person currently or formerly smoked; there was significantly less stigmatization by participants who received the scenario where the person did not smoke. Within negative attributions about lung cancer, the scenarios where the person currently or formerly smoked were comparable and both higher than the scenario where the person did not smoke, suggesting that people with lung cancer who have never smoked may be stigmatized less than those who ever smoked.

Adjusting for the appropriate covariates, the amount of time someone spends concerned about lung cancer was associated with blaming the victim (Aim 3), with the highest stigma mean scores among participants who “worry all of the time”. This suggests that people who smoke with high concern about getting lung cancer may stigmatize people with lung cancer more so than those with less concern, which may reflect attitudes toward their own smoking behavior. Respondents who reported worrying about lung cancer all of the time may regret their smoking and externalize this regret as stigma/blame.

Previous research has suggested that stigma may be a useful tool to move people who smoke toward leaving their stigmatized group (e.g. quitting smoking), if used in the right context^27^. Yet, stigma may inhibit smoking cessation efforts through misreporting of smoking status and social withdrawal^11^. Cessation interventions that address stigma may be efficient in promoting engagement and cessation outcomes. Furthermore, stigma can be a barrier to lung cancer screening, and tailoring screening messages by smoking status may lessen this burden^11^. From a public health perspective, the results of this study support the development of campaigns to reduce interpersonal lung cancer stigma amongst people who currently and formerly smoked^25^. Specific messaging guidelines can include educating the public about all risk factors for lung cancer, explaining the reason that asking ‘Why did you smoke?’ can be hurtful, telling personal stories of people with lung cancer, avoiding an over emphasis on an individual’s smoking status, and educating the public about the powerfully addictive nature of tobacco^28^.

### Limitations

Only people who currently smoked at the time of the study participated; therefore, no comparison could be made with former or never smokers. Although lung cancer concern was measured using a single-item measure, previous literature has validated the use of other single-item cancer concern measures, for example, for breast cancer^29^. The scenarios used in this study were hypothetical and thus might not reflect responses to a real-life situation, though previous studies regarding stigma have used hypothetical measures^30^ and findings reflect the feelings of the respondent. Given the large sample size (n=1419), caution should be taken when interpreting statistical versus practical significance, although the effect sizes found are large. Majority of the sample (74.7%) reported their race as White, which is a limitation of the representativeness of the study. Finally, literature has shown that cancer survivors may be more likely to identify with positive labels compared with negatively toned labels (i.e. victim)^31^. However, this study involved a previously defined framework for lung cancer stigma and the term was not presented to participants of this study.

### Future perspectives

Future research should compare the stigmatization of people with lung cancer of different smoking status by people of different smoking status. Future research should further explore if people with lung cancer who formerly smoked are stigmatized less than those who currently smoke. Also, the discrepancies between studying different perspectives should be explored (i.e. from the point of view of those stigmatizing or being stigmatized). Finally, more research is needed on public health campaigns and messaging guidelines to reduce internalized stigma and the associated negative consequences with regard to people who currently smoke.

## CONCLUSIONS

This study provides a novel perspective to the existing literature on lung cancer stigma. We looked at how stigma differed based on the smoking status of the person with lung cancer, from the viewpoint of those stigmatizing. The theory of the communication management of stigma by Meisenbach^32^ provides stigma management strategies. One of these strategies includes avoiding, which is described as ‘the elimination of the stigma attribute’ that ‘allows the individual to manage stigma by proclaiming the self as ex-stigmatized. This elimination focuses on a change in an individual’s relationship to an accepted stigma^32^. Cessation removes a person from the stigmatized group of people who smoke. We suggest specific messaging guidelines that avoid an over emphasis on an individual’s smoking status, cessation interventions that address stigma, and screening messages tailored to smoking status.

## Data Availability

The data supporting this research are available from the authors on reasonable request.
